# Nutrition Can Help DNA Repair in the Case of Aging

**DOI:** 10.3390/nu12113364

**Published:** 2020-11-01

**Authors:** Julia Kaźmierczak-Barańska, Karolina Boguszewska, Boleslaw T. Karwowski

**Affiliations:** DNA Damage Laboratory of Food Science Department, Faculty of Pharmacy, Medical University of Lodz, ul. Muszynskiego 1, 90-151 Lodz, Poland; julia.kazmierczak-baranska@umed.lodz.pl (J.K.-B.); karolina.boguszewska@umed.lodz.pl (K.B.)

**Keywords:** micronutrients, aging, DNA damage, genome stability, neurodegenerative disorders

## Abstract

Micronutrients such as vitamins and trace elements are crucial for maintaining the health of all organisms. Micronutrients are involved in every cellular/biochemical process. They play roles in proper heart and brain functioning, influence immunological responses, and antioxidant defense systems. Therefore, prolonged deficiency in one or more micronutrients leads to cardiovascular or neurodegenerative disorders. Keeping micronutrients at adequate levels is especially important for seniors. They are prone to deficiencies due to age-associated functional decline and often to a diet poor in nutrients. Moreover, lack of micronutrients has an indirect impact on the genome. Their low levels reduce the activity of antioxidant enzymes, and therefore inhibit the efficiency of defense against free radicals which can lead to the formation of DNA lesions. The more DNA damage in the genetic material, the faster aging at the cellular level and a higher risk of pathological processes (e.g., carcinogenesis). Supplementation of crucial antioxidative micronutrients such as selenium, zinc, vitamin C, and vitamin E seems to have the potential to positively influence the condition of an aging organism, including minimizing inflammation, enhancing antioxidative defense, and limiting the formation of DNA lesions. In consequence, it may lead to lowering the risk and incidence of age-related diseases such as cardiovascular diseases, neurodegenerative diseases, and malnutrition. In this article, we attempt to present the synergistic action of selected antioxidant micronutrients (vitamin C, vitamin E, selenium, and zinc) for inhibiting oxidative stress and DNA damage, which may impede the process of healthy aging.

## 1. Introduction

Malnutrition, according to the definition, is an imbalance at the cellular level between the demand for nutrients and their intake. The fulfillment of nutritional needs supports proper growth and maintenance of the body’s vital functions [1]. Malnutrition should not be associated only with skinny people with an anorectic appearance. It is a common clinical problem, with numerous causes, such as poverty, caring negligence, aging, chronic somatic diseases, or deliberate action to reduce weight. Therefore, it is difficult to estimate its actual scale. According to the WHO, approximately 45% of children’s deaths (under age five) are caused by malnutrition, mainly in destitute and middle-income countries. Interestingly, these countries also have an increasing percentage of overweight and obese children. In that case, eating high-energy but low-nutrient meals results in qualitative malnutrition. The same applies to adults for whom stressful lifestyle and increasingly inappropriate eating habits lead to dietary deficiencies. In obese people, malnutrition can result from a shortage of nutrients, vitamins, and microelements which are necessary for the proper functioning of the body. In addition, low-calorie or elimination diets (such as a vegan diet), if a balanced eating plan is not adhered to, can cause malnutrition by increasing the risk of protein and vitamin deficiencies [2].

One of the more interesting forms of malnutrition is the so-called anorexia of aging, i.e., the loss of appetite associated with aging. It concerns approximately 25% of Europeans over 65 years of age [3]. The risk of anorexia in seniors is higher due to physiological changes associated with aging, coexisting diseases, and medical treatments. Moreover, elderly people often struggle with psychosocial problems such as poverty or social isolation, which strongly predisposes them to loss of appetite. Anorexia is an independent risk factor for death in an older population [4]. As it is associated with qualitative and/or quantitative nutritional deficiencies, immune functions, metabolism, and antioxidative defense systems are weakened. A shortage of polyunsaturated fatty acids (PUFAs), vitamins, micro- and macroelements is partly responsible for geriatric syndromes such as frailty (i.e., drastic functional decline leading to multiorgan impairment). A special model of nutrition for longevity has not yet been identified, but a well-balanced diet with a sufficient quantity of nutrients promotes healthy aging in contrast to malnutrition, which increases susceptibility to disease.

Although aging is a natural process and not a disease, older people are more prone to illness. The feeble and malnourished organism can suffer from, among others, impairment of the immune system. *Inflammaging* (low-grade chronic inflammation) develops with age and may speed up the deterioration processes and worsen other age-related disorders [5,6]. Age-specific conditions also coincide with chronic subliminal inflammation. Fagiolo et al. observed mononuclear peripheral blood cells in the elderly population as compared with healthy young people [7]. The results showed a higher concentration of tumor necrosis factor (TNF-α) and proinflammatory cytokines, interleukin 6 (IL-6) and interleukin 1 (IL-1), during 72 h incubation with the mitogen. Additionally, elevated levels of IL-6 or TNF-α affected nutrition control centers, suppressed appetite, changed sensory sensations, and inhibited muscle protein synthesis [8], all of which could promote the development of anorexia. Subsequently, it could be a cause of inadequate nutrient intake or malfunctioning absorption.

**Figure 1:**
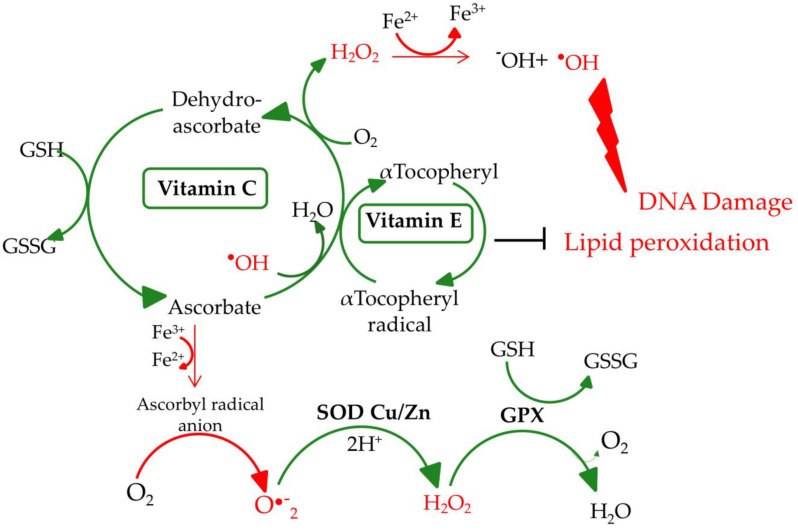
Antioxidant network. SOD Cu/Zn, Cu/Zn superoxide dismutase; GPX, glutathione peroxidase; GSH, glutathione; GSSG, glutathione disulfide [9].

Malnourished older people can have deficits of most micronutrients, including zinc, selenium, vitamin C, vitamin E, riboflavin, electrolytes, and others. Most importantly, micronutrients directly affect (e.g., vitamin C and vitamin E) or indirectly (e.g., selenium and zinc) the activity of antioxidant defense systems (e.g., antioxidant enzymes, superoxide dismutase (SOD), glutathione peroxidase (GPX), and catalase (CAT)) [9]. The proper operation of these antioxidant systems is highly important for the whole organism. They protect the cell against endo- and exogenous pro-oxidative factors, including reactive oxygen species (ROS). The choice of the four presented micronutrients was dictated primarily by their antioxidant properties implemented through antioxidant enzymes. Selenium, in the form of selenocysteine (Sec), is present in the active center of selenoproteins, including GPX, in which the main function is to neutralize H_2_O_2_ and organic peroxides. Vitamin E also neutralizes peroxides and its action is synergistic to vitamin C, selenium, and zinc. Zinc is a component of enzymes from the group of SOD, which catalyzes the dissolution reaction of the O_2_^•−^ to H_2_O_2_ and O_2_. Vitamin C reduces ROS level (i.e., O_2_^•−^, ^•^OH, and ^1^O_2_) but at the same time, it regenerates the oxidized form of vitamin E to its reduced form. Micronutrients selected for this review benefit from the presence of each other and sustain the overall effectiveness of antioxidant defense of the organism. The synergistic effect of the presented microelements on the antioxidant network is illustrated in Figure 1.

**Figure 2:**
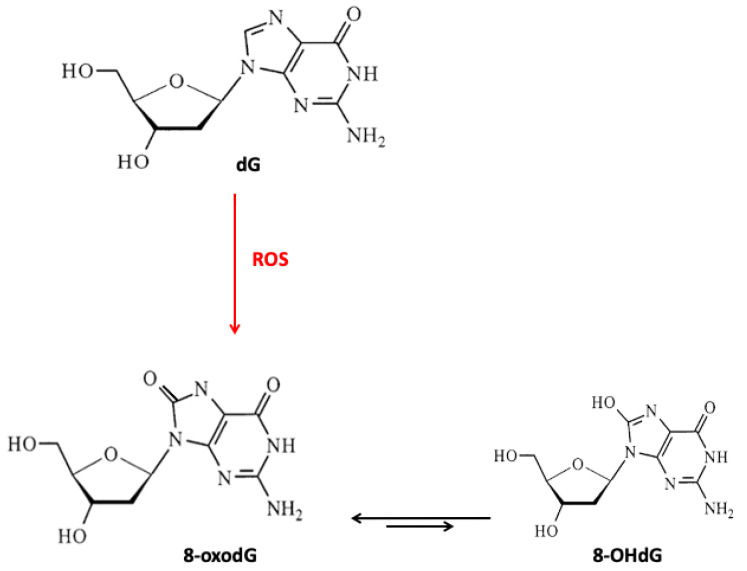
Guanosine and its oxidative modifications. dG, 2′-deoxyguanosine; 8-oxodG, 8-oxo-7,8-dihydro-2′-deoxyguanosine; 8-OHdG, 8-hydroxy-2′-deoxyguanosine; ROS, reactive oxygen species.

The proper operation of antioxidant systems is highly important for the whole organism. Antioxidants protect the cell against endo- and exogenous pro-oxidative factors, including ROS. Their levels increase, especially, in the case of physiological stress, for example, inflammatory processes, malnutrition, and anorexia [10]. In addition, physiological decline connected with age can cause an increase in ROS levels. High ROS levels and lack of nutrients in an older body are even more dangerous as they accelerate aging and the incidence of age-related disorders. ROS formed during physiological reactions are necessary for proper gene expression and cell differentiation but in excess, ROS lead to the formation of DNA lesions and can impact the integrity of the nuclear and mitochondrial DNA (mtDNA). H_2_O_2_ does not pose a direct threat due to its moderate activity, but, in certain conditions, it can be transformed into highly reactive ^•^OH (Figure 1) [11]. Due to its low redox potential, guanine is the most susceptible to oxidation. The major type of guanine lesions is 8-oxo-7,8-dihydro-2′-deoxyguanosine (8-oxodG) and its enol form 8-hydroxy-2’-deoxyguanosine (8-OHdG) formed 10^5^ times per day per cell (Figure 2) [12].

Oxidative lesions are detected and corrected mainly by base excision repair (BER) which is the only mechanism able to correct single nucleotide lesions. DNA repair mechanisms are also affected by nutritional deficiencies which can lead to decreased repair efficiency and subsequent mutations resulting in pathological conditions (e.g., carcinogenesis or neurodegeneration) [13].

This review focuses on selected micronutrients involved in the DNA repair processes and antioxidant protection of the body in the case of malnutrition and anorexia-related deficiencies in the elderly population.

## 2. Biochemical Aspects of Selected Micronutrients

The most common biochemical problems that affect patients with anorexia nervosa are dehydration or electrolyte disturbances due to insufficient supply of micro- and macroelements in the diet. Physiologically, the concentration of K^+^ ions is much higher inside the cell, while the concentrations of Na^+^, Ca^2+^, and Cl^−^ ions are higher in the extracellular space. In anorectic patients, hypokalaemia is a frequent problem. It is dangerous due to the possible consequences such as cardiac arrhythmias, abnormal nerve conduction, or paralysis in striated and smooth muscles, etc. [14]. Moreover, hypophosphatemia has also been observed in patients with an anorexia-related condition, which affects phosphate functions (including ATP synthesis) and impairs glucose metabolism, enzyme phosphorylation, and acid-base management. The biochemical imbalance can lead to the development of, for example, osteomalation, rhabdomyolysis, central nervous system disorders, or hemolysis [15]. Other most important microelements, which regulate the overall well-being of the human organism, are trace elements and vitamins such as selenium, zinc, vitamin E, and vitamin C, which are discussed in this article.

### 2.1. Selenium

Selenium (Se) is present in the active centers of many proteins and enzymes in the form of Sec residues. Selenoproteins play crucial roles in the proper functioning of the whole organism; selenoprotein K (SELENOK, SELK) participates in the construction of protein-protein complexes, selenoprotein M (SELENOM, SELM, SEPM) is involved in the protection of neurons against oxidative stress, and selenoprotein N (SELENON, SELN, SEPN1) is involved in the regeneration of skeletal muscle tissue [16]. Iodothyronine deiodinases (DIO1-3) which are involved in the formation of thyroid hormones are also an example of selenoproteins. Selenium is associated with an immuno-inflammatory and proinflammatory response. Its deficiency in endothelial cells results in reduced production of prostaglandins (PGI2, PGE2, and PGF2α). Selenoproteins also regulate macrophages’ migration and phagocytosis [17].

Selenoproteins have antioxidant properties and are involved in the regulation of the antioxidant defense system. A low level of Se causes reduced cells’ resistance to free radicals. In this context, selenoprotein P (SELENOP, SELP, SEPP1), thioredoxin reductase (TXNRD1, TRXR1), and glutathione peroxidases (GPX) are the most important. SELP acts as an antioxidant, while TRXR1 provides proper cell growth, DNA synthesis, replication, and apoptosis’ inhibition [18,19]. GPX are involved in the protection against oxidative damage by conversion of H_2_O_2_ to water (Figure 1). Interestingly, the level of glutathione (GSH), which is a cofactor for the antioxidant enzymes (GPX and glutathione transferase), is reduced in people suffering from anorexia. Therefore, their ability to detoxify electrophilic metabolites and neutralize ROS is impaired [20]. It can lead to peroxidation of membrane lipids, oxidation of unsaturated fatty acids, reduced membranes’ fluidity/permeability, and, as a consequence, to pathological conditions such as atherosclerosis, diabetes mellitus, or rheumatoid arthritis [21].

Severe selenium deficiency often manifests as cardiomyopathies and heart failures which are often seen in anorexia patients. Kashin–Beck disease is a musculoskeletal disorder with abnormal bone development, growth inhibition, joint pain, and edema with reduced mobility, which is caused by the Se shortage in an unbalanced diet. Therefore, monitoring the selenium level in a patient’s body and fluids can be helpful and is recommended from the therapeutic point of view. Lack of Se can also result in neurological symptoms (e.g., depression), which are often observed in people with anorexia or malnutrition [22,23].

Selenium is considered to be a factor of prolonged life expectancy. Recently, Hammad et al. demonstrated the relationship between Se and replication senescence in human embryonic fibroblasts (WI-38). The authors highlighted that the lack of Se was related to increased ROS levels in aging cells and decreased antioxidant defense (including the activity of selenoproteins) [24]. The addition of selenium increases the number of cell divisions and reduces aging markers (β-galactosidase (SABG) and heterochromatin foci (SAHF)), while its deficiency accelerates senescence and reduces the cell’s proliferative capacity [25]. Mice’s diet enriched with Se enhances the activity/level of SOD, GPX, and total antioxidant capacity (T-AOC) [26]. Selenium may also reduce oxidative stress in peripheral blood lymphocytes, and therefore improves healthy aging [27].

An elderly population usually has reduced selenium levels [28,29]. Adding Se to seniors’ diets may be an important factor in preventing age-related diseases and improving their quality of life (QoL). A study on 347 elders (age > 80) showed that low plasma selenium levels correlated with high levels of IL-6 and C-reactive protein (CRP) [30]. Moreover, all-cause mortality was higher in people with low selenium (≤105.3 µg/L). The authors suggested that higher selenium levels had a positive effect on age-related inflammation. Interestingly, the synergistic effect of Se and vitamin E for quenching free radicals was observed. Patients with selenium-related diseases often have vitamin E deficit (discussed in further sections) [31]. Having in mind that excess of Se can induce adverse effects (diarrhea, fatigue, hair loss, and joint pain) [32], a carefully planned and advised diet enriched in selenium may potentially improve seniors’ QoL.

### 2.2. Zinc

Zinc (Zn) is essential for proper functioning of the cells. It plays an important role in transcription regulation. Zn deficiency often occurs in a malnourished organism and can lead to growth retardation, delayed pubescence, impaired wound healing, dermatitis, decreased appetite, and mental lethargy [33]. Zn is involved in metabolic processes, for example, immune response, as well as neurobehavioral and physical development. Its deficiency impacts antibody production, cytokine production (interleukin 2 (IL-2) and interferon γ (IFNg)), cell signaling, proliferation, and the function of B, T helper, and natural killer (NK) cells [34,35].

Moreover, zinc is present in zinc-finger domains of many proteins such as transcription factors and regulatory proteins. The presence of Zn^2+^ ions is also crucial for the stability of DNA binding proteins because the zinc-finger domain is directly involved in the binding process of the nucleic acid molecule [36]. Moreover, zinc neutralizes the O_2_^•−^ as a component of the Zn/Cu-SOD and is a crucial element (as part of a catalytic domain) of the metalloproteinases [18]. Metallothioenins (MTs) are a family of highly conserved cysteine-rich metalloproteins [37]. MTs have strong antioxidant properties, i.e., they can scavenge ROS and detoxify heavy metals ions [38]. The availability of microelements, such as selenium or zinc, regulates MTs production and cellular accumulation. MTs’ expression increases during stress conditions (e.g., inflammation). It is of interest to describe the so-called redox cycle with MTs. The sulfone group confers redox activity to the Zn-MT complex and can be oxidized and reduced with simultaneous release and binding of Zn in an oxidoreductive environment. Zinc released from MT is available to other molecules. This process is modulated by GSH and glutathione disulfide (GSSG). A more oxidized state results in the release of zinc and a more reduced state promotes MT stabilization [39]. A reduction in oxidized MTs restores its ability to bind Zn. The MT genes have been characterized as one of the few longevity genes. Transgenic mice with *MT* overexpression live longer. Yang et al. observed that their cardiomyocytes inhibited age-related cytochrome C release and generated lower levels of superoxides as compared with control mice. The authors highlighted MTs’ direct impact on cardiac aging and lifespan [40].

Zinc lowers the level of proinflammatory cytokines and markers of oxidative stress. Studies conducted on healthy adults (age 55–87) have shown that monocytic cells of zinc supplemented people generated significantly less TNF. A six-month supplementation led to a significant reduction in TNF levels (1897 ± 1004 pg/mL to 1411 ± 786 pg/mL) as compared with the placebo group (1728 ± 498 pg/mL to 2698 ± 785 pg/mL). Moreover, there was a significant decrease in plasma oxidative stress markers (malondialdehyde (MDA), 4-hydroxyalkenals (HAE), and 8-OHdG) in the supplemented group, with no change in the placebo group (8-OHdG, 0.63 ± 0.16 ng/mL to 0.50 ± 0.14 ng/mL (*p* = 0.030) in the supplemented group vs. 0.66 ± 0.13 ng/mL to 0.68 ± 0.13 ng/mL in the placebo group; MDA + HAE, 1.66 ± 0.343 µmol/L to 1.35 ± 0.18 µmol/L (*p* = 0.0002) in the supplemented group vs. 1.70 ± 0.30 µmol/L to 1.71 ± 0.35 µmol/L in the placebo group) [41]. The authors concluded that zinc, as a non-mutagenic, relatively non-toxic, effective anti-inflammatory and antioxidative agent, could be beneficial for preventing chronic disorders associated with oxidative stress in an elderly population.

### 2.3. Vitamin E

Vitamins E is a group of fat-soluble compounds with strong antioxidant properties as they inhibit lipid peroxidation. Vitamin E (α-tocopherol (α-T) and vitamin E) has a synergistic effect with vitamin C, selenium, and zinc. As a constituent of the cellular membranes, vitamin E is the main antioxidant of PUFA. It inhibits oxidation of cellular macromolecules as donating electron interrupts the chain reaction of phospholipids’ oxidation in membranes at the propagation stage (Figure 1). It can be postulated that by protecting cell membranes, vitamin E delays cellular aging [42] and has a beneficial effect on vascular and cardiac function [43]. Moreover, its moiety is built into the ceramides that are part of the intercellular spaces of the stratum corneum and due to strong antioxidant properties, it protects the epidermis, and therefore increases its resistance to UV radiation [44].

A shortage of vitamin E harms the external cell bilayer and can lead to cancer, cardiovascular diseases, as well as infectious and inflammatory processes. Vitamin E deficiency is well characterized by an isolated lack of vitamin E in ataxia with vitamin E deficiency (AVED). AVED is an autosomal recessive neurodegenerative disorder caused by a mutation in the α-tocopherol protein transfer gene (*α-TTP*) with clinical manifestations being progressive spinocerebellar ataxia, loss of deep sensation (proprioceptivity), and areflexia. However, high doses of vitamin E (800 mg/day) inhibit the symptoms’ progression and may even reverse some neurological symptoms [45]. It the case of anorectic and malnourished people, level of vitamin E is often reduced, for example, due to insufficient intake [46,47]. Vitamin E deficiency may result in neuropathies, as well as progressive necrosis of the nervous system and muscles. Patients with anorexia may also experience cardiovascular complications, arrhythmias, peripheral edema, and even sudden cardiac arrest [48].

On the one hand, there is no consistent evidence that a diet enriched with vitamin E protects against chronic diseases or cancer [49,50]. On the other hand, studies conducted on a population with very low levels of micronutrients due to poor living conditions indicated that vitamin-mineral supplementation (including vitamin E) potentially reduced the risk of cancer [51]. It seems that supplementation is effective in a population with low intake and concentration of antioxidant nutrients such as older people; the potential benefits possibly outweigh the side effects [52].

### 2.4. Vitamin C

It is important to supply this vitamin through food, as the human body cannot synthesize vitamin C (vitamin C, ascorbic acid) due to the lack of L-gulonolactone oxidase. Vitamin C is primarily involved in the synthesis of collagen, catecholamines, and L-carnitine [53]. It is a cofactor for numerous enzymes (e.g., hydrolases, oxygenases, and dioxygenases) and is involved in many metabolic processes (e.g., synthesis of adrenaline from tyrosine). Ascorbic acid, as a water-soluble antioxidant, acts as the body’s primary defense against ROS occurring in the water phase. Vitamin C leads to the formation of well-soluble ferrous salts, by reducing Fe^3+^ to Fe^2+^, which can be more easily absorbed from the gastrointestinal tract [54]. At the intracellular level, ascorbic acid is possibly considered to be an ideal antioxidant, it is present in the cell in the right quantity (it varies in different types of cells, fluids, and tissues [55]), neutralizes a large number of free radicals, and is regenerated to some extent [56]. Vitamin C inactivates free radicals, and thus breaks the oxidative chain. Moreover, it strengthens the action and regeneration of α-tocopherol by reducing its radical formed after the reaction of tocopherol with free radical [42].

The level of vitamin C in the plasma decreases with age [57,58]. On the one hand, clinical studies have indicated a relationship between serum vitamin C levels and the risk of cardiovascular disease (e.g., peripheral arterial disease or stroke). Patients with a low vitamin C level (27.8 µmol/L as compared with a control group 51.7 µmol/L, *p* < 0.0001) have a significantly increased CRP (2.51 mg/L vs. control 4.80 mg/L, *p* < 0.0001) and are at higher risk of developing fatal cardiovascular disease [59]. In addition, serum vitamin C levels are inversely associated with stroke incidence [60]. Similar observations apply to patients suffering from diabetes or hypertension, as their serum vitamin C levels are low [61]. On the other hand, studies have shown that daily vitamin C intake did not reduce serious cardiovascular events, cancer outcomes, or cardiovascular mortality [62,63]. Nevertheless, it seems likely that population groups with low vitamin C status may benefit from additional vitamin C intake [64]. It is especially visible in the case of seniors suffering from anorexia, and therefore with an inadequate intake of vitamin C; the high levels of oxidative damage should be observed in this group.

Moreover, vitamin C is involved in the regulation of gene expression [65]. The authors showed that ascorbate deficiency reduced the expression of the TET1-dependent (methylcytosine oxidase ten-eleven translocation proteins) genes crucial for germline development. Reproductive cells lacking vitamin C have a different gene expression profile than controls because vitamin C is a cofactor of TET hydroxylases (involved in the demethylation of DNA). Reduced expression of *TET* genes accompanies many types of cancers [66], which indicates the role of *TET* genes as tumor suppressors [67]. Vitamin C seems to be required for DNA demethylation, and thus proper epigenetic regulation. Studies have indicated that ascorbic acid is a DNA protector, i.e., a group of healthy subjects showed a significant decrease in the level of 8-oxodG (a marker of oxidative stress) in the plasma and urine after supplementation with 500 mg/day of vitamin C [68]. Additionally, a gene expression analysis has indicated that DNA repair processes were enhanced in cells treated with vitamin C, which we discuss below [69].

## 3. Micronutrients, DNA Damage, and Repair

The human body changes with age. Aging is a set of complex biochemical and physiological phenomena determined by the molecular processes that occur at the cellular level. The aging process is influenced by numerous factors ranging from genetic predispositions to lifestyle choices (e.g., diet), performed kind of work, and living conditions. It is a lifelong process constantly shaped by environmental factors which constitute more than 50% of overall human well-being [70]. Modern awareness about the diet as the major factor influencing our health goes as far back as the 19th century, when the phrase “you are what you eat” originated. However, changes occur at different times and intensities in various organs, and they reach the greatest intensity in old age.

According to one of the theories, aging is associated with the accumulation of oxidative changes in macromolecules and cellular structures [71]. Free radicals in physiological quantity play an important role in cell signaling [72,73] but in excess, they could damage the cell. Oxidation may impair the function of cell membranes and proteins (e.g., enzymes and receptors), or may lead to the formation of DNA lesions. The brain is significantly affected by oxidative damage. Intensive metabolism, high content of fatty acids, and relatively low activity of antioxidant enzymes contribute to age-related neurodegenerative disease [74]. ROS are also generated in mitochondria as a part of the electron transport chain (ETC) when so-called electron leakage occurs. When radicals’ production is too high, the function and structure of mitochondria are disrupted, including the integrity of the cristae and the inner membrane. Hindered mitochondrial function can induce a further increase in oxidative stress and subsequent DNA damage [75]. Moreover, the proximity of the respiratory chain may impair the integrity of mtDNA which leads to a decrease in mitochondrial activity with age [76]. Maintaining stable concentrations of ROS in the cells is a major determinant of longevity and healthy aging. Cellular aging is also associated with extensive and irreversible DNA damage within telomeric or non-telomeric genome sequences [77,78,79].

Fundamental factors of aging include oxidative stress, cellular damage, low effectiveness of damage prevention, and inhibited DNA repair. The oxidative DNA lesions manifest as base modifications, strand breaks, or DNA adducts. The most common markers of oxidative DNA damage are 8-oxodG and 8-OHdG. Wolf et al. showed that older rats had higher concentrations of 8-OHdG as compared with young rats [80]. The level of 8-OHdG increased with rats’ age in their heart, skeletal muscles, liver, peripheral blood, or brain. The most significant increase in 8-OHdG level was observed in the heart and peripheral blood lymphocytes (from 0.157 OD at four months to 0.370 OD at 12 months of age and 0.220 OD at four months to 0.550 OD at 24 months of age, respectively), which authors suggested was related to the DNA repair efficiency. Another study analyzed the level of 8-oxodG in human leukocytes in different age groups in correlation with the concentration of ascorbate in the plasma [81]; the 8-oxodG level increased in leukocytes’ DNA with age. The authors concluded this could have been related to the decrease in antioxidant defense with age (lower ascorbate level).

**Figure 3:**
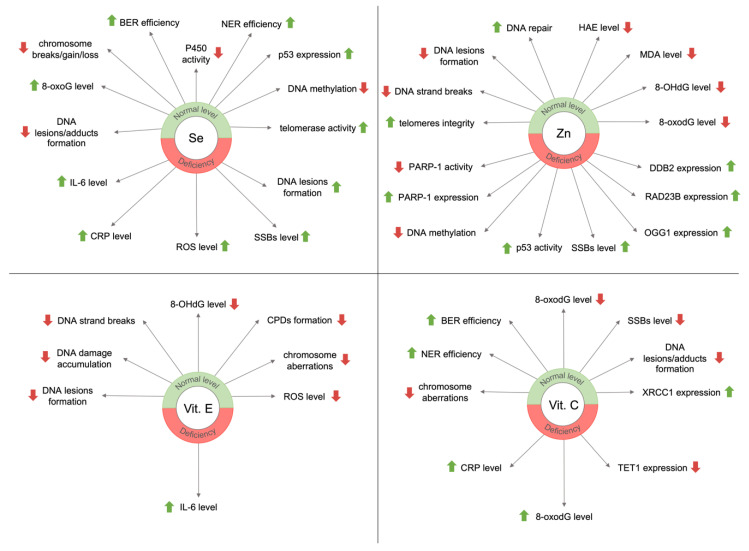
Effects of selected micronutrients on genome stability. BER, base excision repair; NER, nucleotide excision repair; 8-oxoG, 8-oxo-7,8-dihydroguanine; 8-oxodG, 8-oxo-7,8-dihydro-2′-deoxyguanosine; 8-OHdG, 8-hydroxy-2′-deoxyguanosine; SSBs, single-strand breaks; P450, cytochrome P450; IL-6, interleukin 6; CRP, C-reactive protein; ROS, reactive oxygen species; CPDs, cyclobutane pyrimidine dimers; HAE, 4-hydroxyalkenals; MDA, malondialdehyde; PARP-1, poly[ADP-ribose] polymerase 1; OGG1, 8-oxoguanine glycosylase 1; DDB2, damage-specific DNA binding protein 2; RAD23B, RAD23 homolog B; XRCC1, X-ray repair cross-complementation group 1; TET1, methylcytosine oxidase ten-eleven translocation proteins.

For chronic malnutrition, the lack of antioxidants is pro-oxidative, i.e., it increases oxidative stress and impairs ROS neutralization via GSH [82]. Studies have shown that the consumption of antioxidative micronutrients reduced the level of DNA damage or improved DNA repair efficiency. Moreover, micronutrients are important for maintaining genome stability. Their deficiencies can lead to DNA damage formation similar to those resulting from radiation (DNA strands’ or chromosomes’ breaks) [83,84]. Figure 3 presents the main effects of selected micronutrients on genome stability concerning their deficiency or normal level in the body.

### 3.1. Selenium

Selenium is involved in the protection against the negative effects of ROS action. Selenium is a ROS scavenging agent and an element of selenoproteins which catalyzes reactions of ROS removal from an organism. However, it is also known to have an impact on genome stability (Figure 3). It has been shown to inhibit DNA adducts formation with, for example, carboplatin, polychlorinated biphenyl (PCB), or 7,12-dimethylbenz[α]anthracene (DMBA), and lower the number of chromosome breaks, gain, or loss resulting from carcinogens [85]. Moreover, selenium seemed to support the repair of oxidative DNA damage. The potential of repair by an incision was significantly higher in protein extracts from cells pretreated with Se as compared with a control (30% vs. 20% excision, respectively). Mice with Se deficit had upregulated genes induced by DNA damage, which suggested that Se deficiency could be a stress factor for the cell [86].

Another study examined the influence of selenium on the level of DNA damage in a group of 43 people (age 50–75) [87]. Subjects with an initial Se level below the average of 100 ng/mL had higher levels of DNA damage in peripheral blood lymphocytes. The authors suggested that serum Se levels should be kept above 100 ng/mL as DNA damage prevention. Se supplementation is also beneficial in patients with a *BRCA1* mutation [88]. BRCA1 is involved in the repair of DNA double-stranded breaks (DSBs), acts as a tumor suppressor, and maintains genome stability. Urine collected from supplemented (300 µg/day of sodium selenite) *BRCA1* mutation carriers contained a higher level of 8-oxo-7,8-dihydroguanine (8-oxoG, a product of BER system) as compared with a non-supplemented group. The median of 8-oxoG, in the urine samples of *BRCA1* mutation carriers with supplementation, reached 7.75 nmol/mmol creatinine as compared with 5.77 nmol/mmol creatinine in *BRCA1* mutation carriers with no supplementation. Additionally, about a 26% reduction in the 8-oxoG background level in cellular DNA was observed in supplemented patients. These results indicate that Se supplementation enhances the repair of oxidative damage. Selenium can influence gene expression and activate silenced genes (e.g., antioxidant enzymes or tumor suppressors) through epigenetic modulation of histones and DNA in prostate cancer cells (LNCaP). Xiang et al. showed that selenite treatment caused partial demethylation of promoter DNA and re-expression of glutathione S-transferase, decreased overall DNA methylation, and restored expression of the tumor suppressor adenomatous polyposis coli (APC) and cellular stress response 1 (CSR1). The authors concluded that Se could play a role in the chemoprevention of prostate and other cancers through epigenetic regulation of anticancer genes [89].

Seleno-L-methionine (Se-Met) is a naturally occurring selenium-containing amino acid and it appears to selectively regulate the nucleotide excision repair (NER) pathway. Studies on human fibroblasts have shown that pretreatment with Se-Met (10 µL) protected the mouse embryonic fibroblast (MEF) cells from UV-induced DNA damage and induced p53-dependent DNA repair [90]. Similar results apply to human prostate adenocarcinoma cells [91]. Pretreating cell culture with 10 μM of Se-Met protects DNA against damage induced by UVA (50 J/cm) or H_2_O_2_ (200 μM). Studies conducted in hemodialysis patients with chronic kidney disease have shown that the subjects had a lower concentration of selenium than healthy people (40.6 ± 10.4 ng/mL vs. 52.7 ± 9.7 ng/mL, *p* < 0.0001). Moreover, the number of DNA single-strand breaks (SSBs) in white blood cells was significantly higher (DNA damage expressed as the tail moment (0.73 ± 0.84) as compared with the control group (0.25 ± 0.24; *p* < 0.01). After a three-month supplementation (200 μg/day of selenium as Se-rich yeast), 2.6 times lower levels of oxidative damage were observed in hemodialysis patients as compared with a control group [92].

Evidence that an older population needs higher selenium intake also comes from a study on primary human keratinocytes collected from normal skin biopsies [93]. Keratinocytes obtained from older subjects (age 60–70) were more susceptible to UVA-induced damage. Additionally, to inhibit the cytotoxic effect of radiation, the cells required eight times higher doses of Se than those from younger people (age 20–30). Se supplementation may be beneficial for the elderly as it can activate telomerase and p53 expression [85]. Furthermore, selenium inhibits the cytochrome P450 system’s activity (phase I proteins). It converts chemical carcinogens into their reactive forms leading to the formation of various DNA lesions [94,95,96].

Interestingly, studies have shown that selenium deficiency can promote cancer development in humans [97,98]. While Se supplementation in a well-nourished population is rather modest, it may be healthful in older people with low levels of Se [99]. Se deficiency arises from many factors such as improper intake, lack of accompanying nutrients (e.g., methionine), bowel diseases impacting Se absorption, or variations of Se-related genes [85]. Despite plenty of studies about the influence of selenium on DNA damage, DNA repair, and aging, there are no clear guidelines for selenium daily supplementation, because the adequate level of Se is highly individual [100]. Current intake recommendations vary in the range of 25–150 µg/day (depending on sex and the recommending country) [85]. However, it must be taken into consideration that too much selenium can be as damaging as too little and it should be supplemented according to individual needs, especially in an elderly population.

### 3.2. Zinc

Zinc is a crucial element for the overall well-being of the human organism and its genome stability (Figure 3). It is involved in apoptosis, cell proliferation, protection against free radicals, and DNA repair pathways. Zn plays a role as a cofactor of the antioxidant enzymes (e.g., Zn/Cu-SOD) and also of DNA repair-related enzymes such as 8-oxoguanine glycosylase 1 (OGG1), apurinic/apyrimidinic endonuclease (APE), and poly(ADP-ribose) polymerase 1 (PARP-1). Zinc is also a part of a tumor suppressor p53 protein which is responsible for the cell cycle arrest, thus, allowing DNA to repair before replication starts. A lack of Zn upregulates p53 activity (however, 50% Zn depletion results in non-functional p53) and affects DNA repair response [83]. A different study indicated the important role of zinc in the activity of the PARP-1 protein [101]. PARP-1 functions in various repair mechanisms including BER, NER, and DSBs repair [102,103]. It contains the zinc finger motif and is crucial in DNA repair; it detects DNA damage and facilitates the selection of the repair path. Zn deficiency, which is characteristic of an older population, reduces PARP-1 activity, and thus reduces the effectiveness of BER [101]. PARP-1 also has a vital role in inflammatory processes that accompany aging organisms [104].

A shortage of Zn can lead to the accumulation of oxidative DNA lesions. OGG1 and PARP-1 expression levels are higher (*p* < 0.05) in zinc-deficient cells, in addition to a significant increase in DNA strand breaks (*p* < 0.05) [105]. Studies on primary human fibroblasts showed that zinc deficiency induced oxidative stress and DNA damage (SSBs). It also modulated the expression of DNA repair enzymes [106]. Microarray analysis have showed that a lack of zinc affected genes involved in DNA damage, DNA repair, and oxidative stress; two-fold upregulation of damage-specific DNA binding protein 2 (DDB2) and 0.5-fold downregulation of RAD23 homolog B have been observed.

Dietary restriction and repletion of Zn affect DNA integrity. A study on healthy men (age 19–50) showed an increased number of DNA strand breaks during six weeks of Zn restriction. The study proved the importance of dietary Zn for genome stability, because the level of DNA breaks dropped after Zn repletion [107]. After xix weeks of low zinc consumption, DNA damage was significantly increased in the peripheral blood cells (mean tail moment increased by 57%, *p* < 0.05). Interestingly, zinc supplementation reduced DNA damage (mean tail moment decreased by 39.9%, *p* < 0.01). Moreover, a clinical study on 200 patients (age 65–80) showed that Zn supplementation (20 mg/day) improved genome stability and telomeres integrity [108]. After 12 weeks of supplementation, the activity of Cu/Zn SOD in erythrocytes was significantly higher in the Zn group vs. the control group (activity increased by 33.07% in the Zn group, while the placebo group showed only a 2.45% increase (relative to the initial value)). This study also showed a decrease in the micronuclei (MNi) and DNA damage formation as compared with the non-supplemented group (MNi per 1000 binucleates, 6.930 vs. 11.125, *p* = 0.001). Additionally, patients in the supplemented group had a lower level of 8-oxodG in the telomeric regions (8-oxodG/kbp telomere, 6.820 vs. 9.937, *p* = 0.291, respectively). An insufficient dietary intake of Zn results in increased level of oxidative stress and subsequent lesions and it can affect cellular response to those lesions. A deficiency of zinc may increase the incidence of cancer which is especially dangerous for older people as they are already more prone to carcinogenesis. Cancer development and aging are also related to hypermethylation of CpG islands in DNA. Interestingly, studies have shown that Zn depletion led to hypomethylation [109].

Similar to selenium [110], zinc doses should be selected individually, because excess Zn, similar to a deficiency, is harmful and pro-oxidative [111]. Cases of poisoning by drinks containing 2500 mg/L of zinc have been observed. An in vitro study showed that the optimal Zn concentration for DNA damage prevention ranged between 4 and 16 µM [105], while a study on healthy men showed that 11 mg/day of Zn helped to reduce DNA damage [107]. Levels of Zn that are too high can induce DSBs, bases oxidation, and chromosomes’ instability [105]. However, recommendations are yet unclear, as human studies on Zn’s influence on the genome are still lacking. Many variables must be considered while planning supplementation for malnourished patients, especially older patients.

### 3.3. Vitamin E

Vitamin E is the most important agent scavenging lipid peroxyl radicals. It can inhibit lipid peroxidation, H_2_O_2_ action on DNA, and lower oxidative stress resulting from environmental mutagenic factors (e.g., smoke and food additives). The diet of rats enriched with vitamin E (300 mg/kg for six months) significantly decreased the number of chromosomal aberrations in the bone marrow [112]. Other interesting studies have been carried out on animals where α-tocopherol was administered prior to irradiation. In mice pretreated with 100 mg/kg/day of vitamin E, irradiated with 2 Gy, a statistically significant decrease in the incidence of MNi in polychromatic erythrocytes (PCE) was observed [113]. In the supplemented group (200 mg/kg/day of vitamin E) statistically significant protection of the bone marrow against radiation was also detected (expressed as an increase in the PCE/(PCE + NCE) ratio as compared with the positive control, i.e., 7.3% vs. 3.4%, *p* < 0.05). These results suggested that vitamin E could have radioprotective effects. Vitamin E is a well-known ROS scavenging agent that protects against UV-induced DNA damage [114]. It may prevent the formation of cyclobutane pyrimidine dimers (CPDs) developed as a result of UVA in human skin cells. Pre- or posttreatment with vitamin E (0.1 mM) results in lower oxidation and DNA damage. Moreover, studies have shown that α-tocopherol protected the DNA of liver cancer cells from oxidative lesions resulting from ionizing radiation [115]. The level of 8-OHdG increased after irradiation (5 cGy) but the effects were reversed by vitamin E enrichment which indicated its genoprotective properties. The protective effect of α-tocopherol against neurodegeneration in prematurely aging mice has also been described [116]. Xpg-/-mice mimic symptoms of Cockayne syndrome patients, i.e., they are highly sensitive to nutritional deficiencies. The authors suggested that vitamin E supplementation inhibited the accumulation of DNA damage and oxidative stress in liver and brain tissues which both significantly deteriorate with age. Studies have shown that vitamin E reduced the formation of DNA damage such as DNA strand breaks or modifications of 8-OHdG. A study on a group of 21 healthy non-smoking men (age 28.9 ± 1.3) showed that an increase in vitamin E intake by an additional 80 mg/day in a high PUFA diet (15%) decreased DNA damage. A high-fat diet causes lymphocytes to be more prone to DNA strand breakage and an increase in vitamin E intake can probably remove this effect [117].

Vitamin E supplementation for older people must be planned carefully as the proper intake depends on many factors such as background level of vitamin E, its supplemented form, duration of treatment, and possible genetic variations (which may alter vitamin absorption or metabolism) [116]. Different forms of vitamin E can have different impacts on oxidative status in older adults, as the study on 71 patients (age 50–55) showed. [118]. Tocotrienol rich fraction and α-tocopherol were administered for six months and DNA damage level dropped for tocotrienol rich fraction in female subjects after six months. The study showed that the form of vitamin E mattered and could have different effects, which were also dependent on sex. Interestingly, vitamin E (100 µM) has been proven to be potentially beneficial for oncological patients due to its antioxidant action and lack of interference with camptothecin (chemotherapeutic) [119]. Nonetheless, other data have shown no beneficial effect of vitamin E supplementation on cellular DNA damage [120,121]. A positive correlation has been described between serum vitamin E levels and the level of 8-OHdG in peripheral blood lymphocytes in premenopausal non-smoking women (age 45–50) [122]. In another study, healthy men (age 50–70) received 500 mg of vitamin E, but there was no effect observed on micronucleus formation after eight weeks of supplementation [120].

While in vitro studies have proven the likely positive effect of vitamin E on DNA damage (Figure 3), human studies have not fully confirmed it. However, lower levels of circulating α-tocopherol are associated with reduced immune function (increased levels of inflammatory markers) and QoL in the elderly [123]. Among 69 elderly subjects (mean age 78.9) the elevated level of IL-6 was observed and accompanied by a decrease in the concentration of vitamin E (R-(0.277), *p* < 0.01). The results correlated with poor physical and mental health. The authors suggested that insufficient intake of antioxidants (including vit. E) led to reduced QoL and increased the risk of age-related diseases. The influence of α-tocopherol on DNA damage is yet to be confirmed to recommend adequate doses for supplementation in an older population.

### 3.4. Vitamin C

Vitamin C is a crucial antioxidative agent with the ability to enhance genome stability (Figure 3). Studies have shown that its adequate intake could lower the number of chromosome aberrations, DNA adducts, and strand breakage [124]. Insufficient consumption of vitamin C led to an increase in the level of oxidative DNA lesions [125]. Fraga et al. showed that a low/poor dietary supply of vitamin C resulted in a two-fold increase in the level of 8-oxodG in sperm DNA. A different study on 112 patients with coronary artery disease showed that low levels of ascorbate and GSH in peripheral blood lymphocytes were accompanied by more frequent chromosomal aberrations [126].

An interesting study conducted on a group of 139 subjects examined the correlation of air pollution, markers of oxidative DNA damage (including 8-oxodG), and DNA repair gene expression [127]. The authors selected genes coding enzymes involved in the repair of 8-oxodG in BER and non-homologous end-joining (NHEJ), i.e., human 8-oxoguanine glycosylase 1 (*hOGG1*), apurinic/apyrimidinic endodeoxyribonuclease 1 (*APEX1*), X-ray repair cross-complementation group 1 (*XRCC1*), *XRCC4*, *XRCC5*, *XRCC6*, and DNA ligase 4 (*LIG4*). The group of subjects living in a more polluted environment had a lower concentration of 8-oxodG in urine (4.16 vs. 4.99 nmol/mmol creatinine) with simultaneously higher plasma ascorbate levels (11.8 vs. 8.3 mg/L) as compared with subjects living in relatively cleaner regions. The authors speculated that higher plasma levels of ascorbate resulted in higher XRCC1 expression in some people and a later increase in BER efficiency (resulting in lower levels of 8-oxodG in urine), thereby protecting the body from oxidative DNA damage. A different study tested 340 healthy Norwegians for dietary and genetic factors influencing DNA damage and repair capacity [128]. Subjects did not undergo a special diet or supplementation. The Food Frequency Questionnaire and tests of fasting blood samples were used. The levels of DNA strand breaks, oxidized lesions, and the activity of BER and NER were measured. The results showed a significant correlation between diet and level of DNA damage. The quantity of strand breaks and oxidized lesions of purines and pyrimidines was higher when subjects consumed fewer vegetables and fruit. The study also showed that female subjects who consumed less fruit had approximately a 20% higher level of DNA damage. The authors observed, among other things, that NER efficiency was 0.141-fold higher for subjects with a higher level of ascorbate and total DNA damage was 0.037-fold lower.

It seems that antioxidant supplementation is especially important for poorly nourished people [129]. Guarnieri et al. showed that the basic level of DNA repair by an incision in mononuclear blood cells was significantly lower in poorly nourished patients as compared with well-nourished patients. At the same time, poorly nourished people had higher levels of oxidized guanine. Vitamin C supplementation was potentially beneficial, because an increase in DNA repair incision capacity was observed, which was not seen in well-nourished subjects. It is possible that the influence of vitamin C on DNA depends on the preexisting level of this vitamin (the protective effect is observed for >50 µmol/L vitamin C in plasma) and individual level of oxidative stress (resulting from environmental factors such as smoking or exposure to mutagenic chemicals). Moreover, vitamin C should be supplemented for longer periods and together with other antioxidants (e.g., vitamin E) to observe possible positive effects [124].

Oxidative stress is widely recognized as the epigenetic factor of aging. Antioxidative micronutrients play a key role in reducing the inflammatory response associated with poor health outcomes in the elderly population. In addition, enzymatic capacity of cellular antioxidants declines with age, therefore, older people with lower peripheral antioxidant parameters and reduced antioxidant capacity are more susceptible to age-related diseases, disability, weakness, and higher mortality throughout a five-year follow-up [130,131]. These factors explain the increasing trend towards researching the effects of antioxidants on aging and preventing age-related disease.

## 4. Conclusions

Micronutrients are an important part of the antioxidant defense mechanisms. Oxidative metabolism inevitably leads to the production of ROS which can cause further oxidation, particularly of cell membranes and nucleic acids. The cell can counteract oxidative damage with endo- and exogenous antioxidants and repair systems. The damaging potential of free radicals is directly inhibited by the action of ascorbate, tocopherols, or enzyme systems, for example, Zn/Cu-SOD and GPX (dependent on selenium). Therefore, vitamins and trace elements (e.g., selenium and zinc) supplied with the diet are crucial for proper functioning of antioxidant enzymes [9]. In older people, the level of oxidative damage increases, thereby disrupting healthy aging at the molecular level. Shortage of microelements, such as vitamin C, E, zinc, and selenium, makes DNA more susceptible to oxidation. One of the most interesting examples are telomeres, which shorten with age. DNA lesions in their sequence (e.g., guanine oxidation) can result in SSBs or DSBs [132], which increase the risk of age-related diseases such as cancer, cardiovascular, neurodegenerative diseases (e.g., dementia), and diabetes [77]. Anorexia of aging is a state of severe deficiencies of microelements involved in antioxidant protection and maintaining genome stability. It accelerates and aggravates the course of the aging process and seriously disrupts the integrity of genetic information, causing SSBs, DSBs, and oxidative DNA damage. These micronutrient deficiencies can be as harmful as DNA lesions resulting from UV rays and chemical agents’ activity.

Understanding the influence of nutrition on cellular and molecular pathways should enable the development of nutritional strategies for maintaining health and possibly for treating and preventing diseases triggered by dietary deficiencies. The elderly population is particularly vulnerable to various deficits due to reduced intake of food rich in vitamins, micro- and macroelements. Micronutrients are essential for the maintenance of physical and cognitive functions in an aging body. Their insufficient consumption can possibly lead to deterioration of health and general QoL. Anorexia of aging additionally worsens the health condition of older patients, as it exacerbates the natural decrease in micronutrient levels that occur with age [133]. Nevertheless, oral supplementation should be recommended with caution in the elderly population, i.e., only for patients with diagnosed deficiencies, under medical supervision, and for a finite time. A well-balanced diet rich in vegetables and fruit should be the most important part of prophylaxis of age-related diseases such as cardiovascular disease, neurodegenerative diseases, or age-related anorexia, as well as a way to promote healthy aging with a high QoL.
